# Safety and tolerability of DIM-based therapy designed as personalized approach to reverse prostatic intraepithelial neoplasia (PIN)

**DOI:** 10.1186/1878-5085-5-18

**Published:** 2014-10-08

**Authors:** Mikhail Paltsev, Vsevolod Kiselev, Ekaterina Muyzhnek, Vadim Drukh, Igor Kuznetsov, Olga Pchelintseva

**Affiliations:** 1 National Research Centre (NRC ‘Kurchatov Institute’), 1, Akademika Kurchatova pl., Moscow 123182, Russia; 2 Peoples' Friendship University of Russia, Miklukho-Maklaya str. 6, 117198 Moscow, Russia; 3 ZAO ‘MiraxBioPharma’, 12 Kutuzovsky av., 121248 Moscow, Russia; 4 Moscow State Medical Stomatological University (MGMSU), Delegatskaya St. 2/1, 127473 Moscow, Russia

**Keywords:** 3,3′-Diindolylmethane, Infemin, Preclinical trials, Bioavailability, Molecularly targeted treatment, Targeted prevention, Safety, Tolerability, Prostatic intraepithelial neoplasia, Prostate cancer, Personalized medicine

## Abstract

**Background:**

It has been shown previously that novel formulation of 3,3'-diindolylmethane (DIM) substance with high bioavailability (Infemin) inhibits tumor development due to the tumor growth rate reduction in the xenograft model of prostate cancer. Prostatic intraepithelial neoplasia (PIN) is considered to be promising as a personalized and preventive treatment strategy of prostate cancer (PC). We assessed the safety of Infemin in men with PIN and discussed the interim results.

**Materials and methods:**

A total of 14 patients with PIN were enrolled. They were randomized to 900 mg DIM or placebo daily for 3 months. Safety was evaluated by adverse events (AEs), laboratory tests and physical examinations.

**Results and conclusion:**

The trial revealed that Infemin treatment is associated with minimal toxicity and no serious adverse events when administered orally for 3 months. We noted three adverse events including nausea and diarrhea in two patients (14%). Combined 95% confidence interval (CI) was 1.8%–42.8%. Therapy was continued in all cases of adverse events.

Good tolerability of DIM-based formulation allows us to recommend it for further clinical trials among men diagnosed with PIN for its efficacy and long-term safety parameters.

## Overview

Prostatic intraepithelial neoplasia (PIN) is associated with cellular proliferations within the lining of acini, prostatic ducts and ductules. Numerous publications have reported that PIN is a major risk factor for the development of prostate cancer (РС) [1,2]. First of all, PIN histologically has many features in common with РС. Moreover, the increased androgen receptor (AR) activity is present in cases of intraepithelial neoplasia and PC. AR can function as a positive regulator of proliferation and stimulate the development of neoplasia which indicates a direct role of AR in promoting PC [3]. Thus, PIN can be predictive for the development of РС in the future.

There is substantial evidence that hyperplastic processes are associated with exposure to hormones [4]. Androgens stimulate growth, development and functional activity of the normal prostate gland. However, men as they age pass through a shift in hormones due to the influence of external and internal factors. The resulting imbalance changes the functional activity of the prostate, the level of androgens and the status of the androgen receptors.

It is considered that testosterone levels play a major role. The biological activity of testosterone is induced in large part by its conversion to 5α-dihydrotestosterone (5α-DHT) by 5α-reductase. Testosterone and 5α-DHT effects are mediated through binding to the AR. They stimulate proliferation of target cells and induce synthesis of IGF-1, TGFβ and other polypeptide growth factors [5].

Several theories have proposed the role of estrogens in the prostate physiology. It is considered that these hormones directly and indirectly affect growth and differentiation of the prostate. Firstly, they affect cells through interaction with estrogen receptors (ERs). Classical signaling provides binding of estrogen-ER complex to estrogen response elements (EREs) in DNA and activates downstream estrogen responsive genes. Moreover, hydroxylated estrogen metabolites 2-hydroxyestrone/16α-hydroxyestrone (2-OHE1/16α-OHE1) influence the proliferation and induce hyperplasia as a result of the increase in the ratio of metabolites towards more aggressive 16α-OHE1 [6,7].

Nowadays, there are no standard treatments for PIN [8]. Thus, drug development based on substances which target certain pathogenic mechanisms seems promising.

3,3-Diindolylmethane (DIM) is a chemical compound with anticarcinogenic activity [9]. DIM acts as a pure AR antagonist that blocks expression of androgen-responsive genes and inhibits DHT-induced AR translocation and nuclear foci formation [10]. Moreover, it has a pronounced antiestrogenic effect, stimulating the production of 2-OHE1, thus improving the 2-OHE1/16α-OHE1 ratio [11]. Many studies have emphasized that DIM interferes with different signaling pathways. According to experimental data, DIM inhibited growth, angiogenesis and invasion of prostate cancer cells by regulating Akt, NF-kappaB, VEGF and the AR signaling pathway [12]. Another important mechanism of DIM action is the ability to induce apoptosis via Bax-Bcl system [13]. Finally, it was demonstrated that DIM is a selective and potent inhibitor of cancer stem cells [14].

However, DIM substance has one significant disadvantage. It exhibits low bioavailability in target tissues [15-17]. Infemin is a DIM-based pharmaceutical composition with enhanced bioavailability of the active substance due to two components (cod liver oil and polysorbate) that provide increased solubility of DIM [18]. α-Tocopherol acetate (vitamin E) as a free radical scavenger provides storage stability of the drug. This novel formulation of DIM quickly enters the blood and the target organs and reaches high plasma concentrations. The level of DIM in rat blood plasma was about fivefold higher, though the 2,000-fold lower dose was administered, compared to crystalline DIM forms [19].

It has been shown in the xenograft model of prostate cancer that Infemin inhibits tumor development due to the tumor growth rate reduction [20]. Moreover, therapeutic effect lasts for 6 days after the cessation of the Infemin administration. This fact revealed stable positive changes, caused by multiple targeted action of new formulation of DIM.

We place a special emphasis on investigations dedicated to the integrated diagnosis, treatment and prevention of PC in individual patients (predictive, preventive and personalized medicine (PPPM)). The ability to reverse PIN may delay the development of prostate cancer. Thus, we identify targets due to determination of signs of the disease at the early stage. Accurate definition of risk groups allows us to select individuals who are candidates for prevention strategies. We suggest that the strategy of treating PIN by addressing key signaling pathways with DIM-based agents will be effective for PC prevention.

A double-blind, randomized, placebo-controlled, prospective phase Ib clinical trial was performed to determine the safety and tolerability of a novel DIM-based drug.

## Methods

### Drug compounds

Infemin consisted of 3,3′-diindolylmethane (150 mg) and organic components (cod liver oil, polysorbate 80 and α-tocopherol acetate (vitamin E)) providing high bioavailability and storage stability of the drug [18].

### Participants and treatments

Fourteen patients (ages between 18 and 60 years) with histologically verified PIN, serum PSA < 10 ng/mL and residual urine volume less than 150 mL were enrolled. Patients should not have prior therapy of chronic prostatitis within 1 month or therapy of prostatic hyperplasia and prostatic intraepithelial neoplasia within 3 months before starting study treatment. Exclusion criteria included also treatment by any investigational drug proceeding within 30 days of randomization. Patients who had a prostate cancer or another tumor, previous surgery for pelvic organ, acute urinary tract infection, urinary retention, neurogenic bladder dysfunction, stones and diverticula, urethral stricture, bladder neck sclerosis and a flow rate that is less than 5 ml/s were also excluded. Other exclusion criteria were addiction or chronic alcoholism, psychiatric disorders and cardiac disease.

Patients underwent clinical screening within 1 month before baseline. Then, they were randomized to receive 900 mg (three capsules twice a day) DIM or placebo daily. Subjects were evaluated for 3 months. If serious adverse events are not present, patients will continue to take Infemin and participate in the trial for 12 months.

It was planned that adverse events (AEs) were assessed through physical examinations at clinic every 3 months (12-month regimen). Every visit usually lasted 1–5 days. At visits, clinical laboratory tests such as standard blood and urine chemistries were performed. Measurement of vital signs, namely systolic blood pressure (SBP), diastolic blood pressure (DBP), respiratory rate (RR), heart rate (HR) and electrocardiography were also monitored. Patients underwent prostate biopsy every 6 months (12-month regimen).

This study was reviewed and approved by the local ethic committee of the Peoples' Friendship University of Russia. Written informed consent was obtained from all patients.

### Statistical analysis

Adverse events were tabulated and rated for severity (mild, moderate and severe). The experimental data were treated statistically using program package Statistica 8.0 (StatSoft, Inc., Tulsa, OK, USA). Statistical tests were two-sided with a significance level of 0.05. The 95% confidence interval was computed using the Clopper-Pearson method. Statistical dependence between the presence of adverse events and group of study was analyzed by chi-square exact test (*χ*^2^).

## Results

We evaluated interim data from the trial of Infemin safety and tolerability profile. Fourteen patients were administered an oral daily dose of Infemin or placebo. All patients in each group completed the study.

There were three mild adverse events which occurred in 2 out of 14 men diagnosed with PIN and no serious adverse events. One patient experienced nausea (AE was repeated twice). After 3 months, one subject receiving the test formulation reported diarrhea. Thus, the most common adverse events were gastrointestinal in nature. These side effects were mild and transient.

All adverse events in these subjects are listed in Table 1.

**Table 1:** Number and percentage of patients reporting adverse events in placebo-controlled clinical trial of Infemin

**Visit**	**Reporting adverse event**	**Group**		**Total**
		**Infemin**	**Placebo**	
**1 (0 day)**	None	8 (89.0%)	5 (100.0%)	13 (93.0%)
	Nausea	1 (11.0%)	0 (0.0%)	1 (7.0%)
**2 (90th day)**	None	7 (78.0%)	5 (100.0%)	12 (86.0%)
	Nausea	1 (11.0%)	0 (0.0%)	1 (7.0%)
	Diarrhea	1 (11.0%)	0 (0.0%)	1 (7.0%)

Patients who received the placebo did not report any side effects. In both groups, there were no reported serious adverse events or toxicities.

The value for the *χ*^2^ after 3 months was 0.157. Thus, we have found no correlation between AEs and group of study.

The vast majority of patients (12 out of 14) did not report any drug-related adverse events (86% versus 100% for placebo). A confidence interval shows the range within which the true effect is likely to lie. For nausea, the 95% confidence interval (Clopper-Pearson method) runs from 0.2% to 33.9%, and for diarrhea, the 95% confidence interval runs from 0.2% to 33.9%. The combined confidence interval for AEs (95% CI) was 1.8%–42.8%.

Patients ingesting Infemin did not show any clinically significant changes in various clinical and biochemical parameters. However, it should be noted that results of the study are interim, and there are missing values for a series of indicators (total protein, albumin, creatinine, glucose, total bilirubin, ALT, AST). Thus, we were not able to evaluate change dynamics of these parameters from screening to the latest visit.

During the study, we have not observed serious adverse events, deaths or adverse events that led to discontinuation of the investigational drug. The intensity of adverse events in this trial was approved by investigators as non-significant. All AEs were considered to be probably unrelated to the experimental drug.

Symptoms caused by drug therapy have been treated in two out of three cases (one patient with nausea) to eliminate or reduce the AEs. After treatment, the signs and symptoms of nausea have disappeared.

In all cases of adverse events, therapy was continued. Thus, we suggested that the drug Infemin is well tolerated.

## Discussion

This phase Ib study of Infemin was performed after preclinical studies suggested tolerability and protective effect of orally administered DIM formulation which demonstrated anticancer activity [21].

Safety of DIM was confirmed in several clinical trials. Accordingly to Reed et al., a single dose of BR-DIM (pharmaceutical composition BioResponse-DIM which contains pure DIM, microdispersed in spray-dried starch particles) at 200 mg in healthy subjects, including men was well tolerated [17].

BR-DIM was also well tolerated by prostate cancer patients in investigation of Heath et al. [15]. The most common toxicities reported were diarrhea and hyperglycemia with no significant changes observed with other laboratory values. Oral BR-DIM at 2 mg/kg/day for 12 weeks also had no significant toxicity (two subjects complained of nausea) [22].

Our findings indicate a good tolerability profile of Infemin. Based on our clinical experience, side effects of gastrointestinal complaints (nausea and diarrhea) were the most commonly reported adverse events that appeared during the treatment. All AEs were considered unrelated to the investigational drug or probably unrelated. However, these side effects were likely related to the cod liver oil component. In spite of this, cod liver oil is a necessary component of formulation which provides high bioavailability of active DIM substance. This phase Ib trial includes only a small number of patients and was exploratory. We plan to present final clinical trial data in the first calendar quarter of 2015.

As men examined in this study are at high risk for prostate cancer, our findings are of particular interest. Infemin treatment may be considered as a novel approach for preventive therapy of PC in personalized medicine. We confirmed good tolerability of novel DIM-based formulation in short-term group. However, efficient Infemin treatment of PIN requires long-term therapy (for 1 year). Thus, Infemin will be investigated during further clinical trials among men diagnosed with PIN for its efficacy as well as long-term safety parameters.

## Abbreviations

2-OHE1/16α-OHE1: 2-hydroxyestrone/16α-hydroxyestrone; DIM: 3,3′-diindolylmethane; 5α-DHT: 5α-dihydrotestosterone; AEs: adverse events; ALT: alanine transaminase; AR: androgen receptor; AST: aspartate transaminase; BR-DIM: BioResponse-DIM; ER: estrogen receptor; EREs: estrogen response elements; PC: prostate cancer; PIN: prostatic intraepithelial neoplasia.

## Competing interests

The authors declare that they have no competing interests.

## Authors’ contributions

MP and VK participated in the design and coordination of the study. EM participated in the design of the study and drafted the manuscript. VD participated in the conduction of clinical trial and drafted the manuscript. IK participated in the conduction of clinical trial and drafted the manuscript. OP participated in the examination of patients and in data analysis and drafted the manuscript. All authors read and approved the final manuscript.
